# Population normative data for three cognitive screening tools for older adults in sub-Saharan Africa

**DOI:** 10.1590/1980-57642021dn15-030005

**Published:** 2021

**Authors:** William Keith Gray, Stella-Maria Paddick, Adesola Ogunniyi, Olaide Olakehinde, Catherine Dotchin, John Kissima, Sarah Urasa, Aloyce Kisoli, Jane Rogathi, Declare Mushi, Akindele Adebiyi, Irene Haule, Louise Robinson, Richard Walker

**Affiliations:** 1 Northumbria Healthcare NHS Foundation Trust, Department of Medicine, North Tyneside General Hospital, North Shields, United Kingdom.; 2 Newcastle University Institute of Population Health Sciences and Clinical and Translational Medicine, Framlington Place, Newcastle upon Tyne, United Kingdom.; 3 University of Ibadan, Department of Medicine, Ibadan, Oyo State, Nigeria.; 4 Hai District Hospital, District Medical Office, Boman’gombe, Kilimanjaro, Tanzania.; 5 Kilimanjaro Christian Medical University College, Department of Medicine, Moshi, Tanzania.

**Keywords:** cognition, screening, Tanzania, Nigeria, sub-Saharan Africa, education, cognição, programas de rastreamento, Tanzânia, Nigéria, África Sub-Saariana, educação

## Abstract

In sub-Saharan Africa (SSA),cognitive screening is complicated by both cultural and educational factors, and the existing normative values may not be applicable. The Identification of Dementia in Elderly Africans (IDEA) cognitive screen is a low-literacy measure with good diagnostic accuracy for dementia.

**Objective:**

The aim of this study is to report normative values for IDEA and other simple measures [i.e., categorical verbal fluency, the Consortium to Establish a Registry for Alzheimer’s Disease (CERAD) 10-word list] in representative community-dwelling older adults in SSA.

**Methods:**

Individuals aged ≥60 years resident in 12 representative villages in Kilimanjaro, Tanzania and individuals aged ≥65 years resident within three communities in Akinyele Local Government Area, Oyo State, Nigeria underwent cognitive screening. The normative data were generated by the categories of age, sex, and education.

**Results:**

A total of 3,011 people in Tanzania (i.e., 57.3% females and 26.4% uneducated) and 1,117 in Nigeria (i.e., 60.3% females and 64.5% uneducated) were screened. Individuals with higher age, lower education, and female gender obtained lower scores. The 50th decile values for IDEA were 13 (60–64 years) *vs*. 8/9 (above 85 years), 10–11 uneducated vs. 13 primary educated, and 11/12 in females vs. 13 in males. The normative values for 10-word list delayed recall and categorical *verbal fluency* varied with education [i.e., *delayed recall* mean 2.8 [standard deviation (SD) 1.7] uneducated vs. 4.2 (SD 1.2) secondary educated; verbal fluency mean 9.2 (SD 4.8) uneducated vs. 12.2 (SD 4.3) secondary educated], substantially lower than published high-income country values.

**Conclusions:**

The cut-off values for commonly used cognitive screening items should be adjusted to suit local normative values, particularly where there are lower levels of education.

## RESUMO

Na África Subsaariana (ASS), a triagem cognitiva é complicada por fatores culturais e educacionais, além dos valores normativos existentes poderem não ser aplicáveis. O rastreio cognitivo *Identification of Dementia in Elderly Africans* (IDEA) é uma medida para níveis baixos de alfabetização com boa acurácia diagnóstica para demência.

**Objetivo:**

Relatar os valores normativos para a IDEA e outras medidas simples (fluência verbal categórica, a lista de 10 palavras do *Consortium to Establish a Registry for Alzheimer’s Disease* (CERAD) em idosos residentes na comunidade, representativos da ASS. Métodos: Indivíduos com idade ≥60 residentes em 12 comunidades representativas em Kilimanjaro, Tanzânia e indivíduos com idade ≥65 anos residentes em três comunidades na área governamental de Akinyele, Estado de Oyo, Nigéria, foram submetidos à triagem cognitiva. Os dados normativos foram gerados por faixas etárias, sexo e escolaridade.

**Resultados:**

Um total de 3.011 pessoas na Tanzânia (57,3% mulheres, 26,4% sem educação) e 1.117 na Nigéria (60,3% mulheres, 64,5% sem educação) foram examinadas. Os indivíduos com idade mais alta, menor escolaridade e mulheres obtiveram escores mais baixos. Os valores do percentil 50 para a IDEA foram 13 (60–64 anos) *vs.* 8/9 (85+ anos), 10–11 para analfabetos *vs.* 13 com educação primária e 11/12 em mulheres *vs.* 13 em homens. Os valores normativos para a evocação tardia da lista de 10 palavras e a fluência verbal categórica variaram com a educação (evocação tardia 2,8 (SD 1,7) para os sem educação, *vs.* 4,2 (SD 1,2) para com educação secundária; fluência verbal 9,2 (DP 4,8) para os sem educação vs. 12,2 (SD 4.3) para os com ensino médio, substancialmente inferior aos valores publicados em países de alta renda.

**Conclusões:**

Os valores de corte para testes de triagem cognitiva comumente usados devem ser ajustados para se adequar aos valores normativos locais, particularmente em níveis baixos de educação.

## INTRODUCTION

The prevalence of dementia in older adults living in sub-Saharan Africa (SSA) is considered to be similar to that seen in high-income countries (HICs).^1^ Furthermore, as the demographic transition continues, the absolute numbers of people living with dementia in SSA are projected to increase substantially.

The diagnosis of dementia can be challenging in low- and middle-income country (LMIC) settings, in part due to the lack of validated and locally normed culturally appropriate measures.^2,3,4^ Tools developed and normed in other settings have well-recognized limitations when used in SSA, leading to high false-positive rates.^5,6^ Educational and cultural differences impact the performance on cognitive screening measures worldwide,^2,7,8^ and this impact appears greater in settings where the levels of illiteracy are high among elders. Specifically, tools that are widely used elsewhere such as the Montreal Cognitive Assessment (MoCA) and Mini-Mental State Examination (MMSE) appear educationally biased and over-report dementia in low-literacy settings in SSA.^9,10^ Most existing SSA epidemiological studies utilize the forms of the Community Screening Interview for Dementia (CSI-D), a fairly lengthy instrument designed to incorporate a cognitive assessment and an informant interview.^1,11^ Although the CSI-D was designed for lower-literacy settings and has been used in urban and rural LMIC settings worldwide, educational bias is evident when used in some SSA settings, with substantial false-positive rates compared with the DSM criteria for dementia and the prevalence of screen-positive dementia of up to 20%.^6,12^ Tools specifically designed for SSA include the Test of Senegal, though this is relatively lengthy and lacks external validation.^13^

Early diagnosis of dementia is beneficial for people with dementia and their carers,^14^ and in SSA, the low-cost interventions for dementia are beginning to be available.^15,16^ The detection of mild cognitive impairment (MCI), a potentially reversible condition, requires the appropriate normative data for diagnosis. This is currently challenging in SSA. Although the accurate epidemiological data on dementia has recently become available in SSA, allowing policy-makers to plan for this growing population,^1^ the data for MCI are more limited. The cognitive metrics are also needed to support the etiological and interventional studies.^17,18^

The Identification of Dementia in Elderly Africans (IDEA) cognitive screen is a brief, multi-domain screening tool developed by our team in rural Tanzania to address the issues of educational bias observed in other cognitive screening measures used in SSA.^19^ Subsequent external validations have taken place in a hospital and community settings in Tanzania and Nigeria for cognitive impairment, dementia, and delirium,^20,21,22^ and it appears not to be significantly educationally biased. A cut-off value of 7/15 appears most accurate in identifying dementia in Tanzania and Nigeria. The IDEA cognitive screen was developed in part from the CSI-D and includes two items that are commonly used worldwide, namely, categorical verbal fluency (i.e., animal naming) and the Consortium to Establish a Registry for Alzheimer’s Disease (CERAD) 10-word recall list of high-frequency words.^23^ The LMIC normative values for the 10-word list and animal naming have been published, but the existing data largely exclude SSA.^24,25^

Within SSA, the majority of studies of dementia have taken place in Nigeria, a West African country with a population aged 60 years and above of 5.1% and life expectancy at age 60 of 13.9 years. More recent studies have taken place in Tanzania, in East Africa (i.e., a population aged 60 and above, 4.6%, life expectancy at age 60, 17.5 years).^26^

The aim of this study is to present the normative data from population samples of older adults in Tanzania and Nigeria for the full IDEA cognitive screen and extract the CERAD 10-word list (i.e., list-learning and delayed recall) and categorical verbal fluency (animal naming) by age and educational level. The motivation of the authors to complete this study was to develop the previous collaboration on the development of screening tools for dementia within the IDEA study and consider the evident research gap on the identification of MCI, given the educational bias noted for other tools. We aimed to provide the data to inform researchers and clinicians assessing cognition in older adults in similar settings in SSA. The 10-word list and animal naming tests were chosen as they are brief tools shown not to be educationally biased in other LMIC settings and with potential to be less affected by floor and ceiling effects in this population.

## METHODS

### Ethics and consent

The ethical approval was granted by the Tanzanian National Institute of Medical Research and the Kilimanjaro Christian Medical University College Research Ethics Committee in Tanzania and by the University of Ibadan Research Ethics Committee and Ethical Review Committee of the Oyo State Ministry of Health, in Nigeria.

Each participant was given verbal information about the study and allowed to ask questions. Written informed consent was obtained by signature or thumbprint, depending on literacy status. An assent was sought from a close relation if the participant was unable to give consent, due to cognitive impairment.

### Setting

The representative community samples of older adults were recruited from Tanzania, East Africa and Nigeria, West Africa (i.e., from LMIC).^27,28^

### Tanzania, East Africa

The Tanzanian data were collected in the Hai District, Kilimanjaro. Hai is a rural region functioning as a demographic surveillance site (DSS) for over 20 years.^29^ Most of the population (i.e., currently approximately 180,000) identify with the Chagga tribe, with smaller numbers of Pare and Maasai groups represented. The predominant occupation among older adults is subsistence farming. Twelve representative villages were selected from 80 within Hai to represent highland, mid-zone, and lowland populations on the mountainside and the differing socioeconomic status. At the village level, socioeconomic status generally increases with increasing soil fertility and rainfall, which are found in areas of higher altitude.

All individuals aged ≥60 years and ordinarily resident within the 12 selected villages were approached for inclusion. The cut-off age of ≥60 years was selected to reflect the compulsory retirement age for public service workers in Tanzania.^30^ The data collection took place between March 9 and July 28, 2018, alongside a house-to-house census, ensuring the population was accurately identified.

### Nigeria, West Africa

In Nigeria, all individuals aged ≥65 years resident within three selected communities in Akinyele Local Government Area, Oyo State (i.e., Ijaiye, Alabata, and Olorisa-Oko) were approached for inclusion from February 15 and October 24, 2018. The communities selected were rural/semi-urban, and the sample size was based on previous dementia prevalence estimates to yield a population of approximately 100 people with dementia. The cut-off age of 65 years coincides with the Nigerian National Population Commission definition of “older persons”^31^ in Akinyele is semi-urban with a population of 211,811,^32^ the majority of whom farm cocoa, maize, and cocoyams. Most identified with the Yoruba tribe and both Yoruba and English are widely spoken.

### Demographic data (age and educational background)

Educational background was measured with the years of education and categorized into no formal education, elementary school (1–4 years), primary school (5–7 years), secondary school, and post-secondary education. Literacy was assessed through self (or informant) reported ability to read and write a simple note currently or previously.

At both sites, age was calculated from the year of birth (i.e., when known) and triangulated from historical events, age at marriage, and the year of birth of first child. This method is well established in both Tanzania and Nigeria^33,34^ and validated to be accurate within 3 years in older individuals where the year of birth is unknown.^35^

### Cognitive screening

The IDEA cognitive screen is scored 0–15 (i.e., maximum) and includes abstraction, orientation, long-term memory, categorical verbal fluency, verbal delayed recall, and visuospatial construction. No items require reading, writing, drawing, or calculating. Previous external validation has established the following cut-offs: 0–7 “probable dementia,” 8–9 ‘possible dementia,” and 10–15 “no dementia”^19^ and the AUROC for dementia or major cognitive impairment in hospital patients aged 60 and above of 0.919 in Tanzania and 0.990 in Nigeria.^20^

The CERAD 10-word list is the delayed recall item of the IDEA and has been used in LMICs as part of the 10/66 dementia research group protocol.^36^ A high-frequency word list is read three times with the person screened and requested to list all words immediately recalled over three trials. Following the three trials, there is a delayed recall task. The delayed recall score is included within the IDEA cognitive screen. The mean delayed word-list recall performance appears broadly similar in older people in diverse LMIC settings.^24^

Categorical verbal fluency was measured through animal naming in 1 minute. A standard instruction was given as follows: “please mention as many animals as you can” with a qualifying statement “this means any animal, living in any place.” Screeners were instructed to encourage participants by nodding and saying, “please continue, as many as you can” and issued with a stopwatch to aid accurate timing. Acceptable responses were agreed and harmonized during training. Only one example of each animal was permitted, and different colors or baby and adult versions of the same animal (e.g., puppy and dog) were classified as repetitions.

Within the IDEA cognitive screen, abstraction is assessed by asking “please tell me, what is a bridge?”. If the person appears unsure, the assessor can prompt like “Imagine that I am a small child and I have never seen a bridge, how will you explain it?”. Visuoconstruction is assessed through a matchstick construction task where the person is asked to replicate a four-matchstick shape of a rake after the demonstration by the examiner.^19^

### Cognitive screening – training and harmonization

In Tanzania, cognitive screening was completed by census enumerators,^29^ a well-established epidemiological research role supervised by the District Medical Office.^29^

Enumerators attended a 2-day workshop where instructions were evaluated for clarity, and screening was calibrated through a role-play with older locally resident volunteers. For the matchstick construction task, enumerators were trained in standardized scoring^37^ and took a photograph of the completed task for external verification.

In Nigeria, screening was completed by four community healthcare workers who were previously trained and certified in these procedures during an earlier epidemiological study.^38^ For this study, further refresher training was provided by a senior specialist nurse (O.O.).

## STUDY PROCEDURES

The IDEA cognitive screen was administered using tablet-based forms developed by using the Open Data Kit (ODK) software (https://opendatakit.org) with the data collection options in Swahili, English, and Yoruba. For each cognitive task, standard instructions were read aloud by screeners. In Tanzania, instructions were given in Swahili, but enumerators (i.e., all of whom were bilingual) were able to translate these to the local tribal language where necessary.

People were screened in their own homes or at a local health or community center, depending on their preference. The data were uploaded from both sites weekly to a secure, encrypted server, held by the Kilimanjaro Clinical Research Institute, Moshi, Tanzania, and quality checked by the study statistician.

### Inclusion criteria

All consenting village residents meeting the age criteria were screened. The two-stage epidemiological surveys of dementia took place at both sites, alongside this study, meaning that a proportion of those meeting dementia criteria were subsequently clinically assessed and received a diagnosis. The diagnosis of dementia following clinical assessment in the second stage of the study was not in the exclusion criteria. Those not stratified to the second stage of the prevalence survey would be very unlikely to receive a diagnosis of dementia from another source, and therefore, within our study design, it would not be possible to accurately exclude all individuals with dementia. Inclusion of all individuals in the “real-world” conditions was felt to be more representative of the situation in rural clinical practice in SSA, and therefore, all residents were included irrespective of subsequent diagnosis.

### Statistical analysis

The statistical analyses were supported by IBM *Statistical Package for the Social Sciences* (SPSS) software for Windows version 23 (IBM Corp, Armonk, NY, USA). The descriptive statistical analysis used standard summary measures with the normally distributed data reported as mean and standard deviation (SD) and with the non-normally distributed data reported as median and interquartile range. Scores for the IDEA cognitive screen total, animal naming, and immediate and delayed recall were reported by deciles where possible and quintiles where deciles were not possible due to the small number of data points within certain categories.

Age was categorized into the 5-year age bands, and education was categorized into “no education,” “elementary school,” “primary school,” and “secondary school and above.” The cognitive scores for those subsequently diagnosed with dementia were analyzed separately. To determine the effect of age, education, and gender on sub-scores, a logistic regression model was constructed. Education was dichotomized into “some formal education” and “no formal education” categories, since this categorization is known to have the greatest impact on cognitive scores in low literacy settings,^39^ and allows a comparison with previous work in these and other LMIC settings.^40^

## RESULTS

In Tanzania, the census population of the 12 villages was 28,236, of whom 3,122 (11.1%) were aged ≥60 years and 3,011 (96.4%) consented to screening. In Nigeria, 1,132 people were eligible for screening, 15 did not consent, and the data were available for 1,117 people.

A total number of participants in each age band, by sex, and in each educational category are shown in Table 1 for Tanzania and Nigeria, together with the decile scores for the IDEA cognitive screen. Scores were progressively lower in higher age bands, with lower education levels, and in females.

**Table 1.** Deciles for the total Identification of Dementia in Elderly Africans cognitive screen score by the categories of age, sex, and education in Tanzania and Nigeria.

Tanzania	10th	20th	30th	40th	50th	60th	70th	80th	90th
Age									
60–64 years (n=775)	10	11	12	13	13	14	14	15	15
65–69 years (n=662)	9	11	12	12	13	13	14	14	15
70–74 years (n=513)	7	9	10	11	12	13	13	14	15
75–79 years (n=437)	6	8.6	10	11	12	12	13	14	14
80–84 years (n=299)	4	6	8	9	10	11	12	13	14
85 years and above (n=365)	0.6	4	5.8	7	8	10	11	13	14
Sex									
Female (n=1,726)	5	8	10	11	12	13	13	14	15
Male (n=1,285)	7	10	11	12	13	13	14	14	15
Education									
No formal education (n=794)	3	6	7	9	10	11	12	13	14
1–4 years of primary school (n=1,033)	7	9	10	11	12	13	13	14	15
5–7 years of primary school (n=1050)	9	11	12	13	13	14	14	14.8	15
Secondary school or higher (n=142)	11	13	13	14	14	15	15	15	15

Nigeria	20th	30th	40th	50th	60th	70th	80th	90th
Age									
65–69 years (n=378)	8	10	11	12	13	13	14	15	15
70–74 years (n=240)	7	9	10	12	12	13	14	15	15
75–79 years (n=139)	6	8	9	10	11	12	13	14	15
80–84 years (n=173)	4	6	8	10	11	12	13	14	15
85 years and above (n=159)	3	5	7	8	9	10	12	13	14
Sex									
Female (n=674)	5	7	9	10	11	12	13	14	15
Male (n=443)	8	10	11	12	13	13	14	15	15
Education									
No formal education (n=721)	5	7	8.6	10	11	12	13	14	15
1–4 years of primary school (n=147)	8	10	11	12	12	13	14	14	15
5–7 years of primary school (n=161)	9	11	12	13	13	14	15	15	15
Secondary school or higher (n=88)	10	12	13	13	14	14.4	15	15	15

Bold values represent deciles.

Given the similarities at both sites, the two datasets were combined for the subsequent analysis. Those presented within the age category of 60–64 originate wholly from the Tanzanian site. Tables 2 and 3 show the quintile scores for the total IDEA cognitive screen, delayed recall, animal naming, and immediate recall (i.e., sum of word list trials 1–3), respectively. All three measures show lower scores in those with lower education level and greater age. Once the effect of age and education is removed, any association with sex appears to be small.

**Table 2.** Quintiles for the total Identification of Dementia in Elderly Africans cognitive screen score by the combined categories of age, sex, and education.

Sex	Age	No formal education					Formal education				
		Number	20th	40^th^	60th	80th	Number	20th	40th	60th	80th
Females	60–64 years	85	8	11	12	14	389	12	13	14	15
	65–69 years	237	8	11	12	14	347	11	12	13	15
	70–74 years	208	7	9	12	14	198	9	11	13	14
	75–79 years	188	6	9	11	13	149	9	11	13	14
	80–84 years	196	5	8	11	12	91	7	9	12	14
	85 years and above	247	3	6	9	12	65	4	7	9.6	13
Males	60–64 years	19	6	8	12	14	310	12	13	14	15
	65–69 years	50	9	11	13	14.8	366	11	13	14	15
	70–74 years	57	9.6	12	13	15	290	10	12	13	15
	75–79 years	55	7	10	12	14	184	10	12	13	14
	80–84 years	55	5.2	8.4	11	13.8	130	8	10	13	14
	85 years and above	110	5	8.4	11	14	102	6.6	9	12	14

Bold values represent quintiles.

**Table 3.** Quintiles for the Consortium to Establish a Registry for Alzheimer’s Disease word list such as immediate recall, delayed recall, and animal naming all by the combined categories of age, sex, and education.

CERAD immediate recall word list-learning											
Sex	Age	No formal education					Formal education				
		Number	20^th^	40th	60th	80th	Number	20th	40th	60th	80th
Females	60–64 years	85	9	12	14	18	389	12	14	16	19
	65–69 years	237	12	14	16	18	347	11	13	16	19
	70–74 years	208	10	12	15	17	198	10	12	14	17
	75–79 years	188	8	11	13	16	149	10	12	14	17
	80–84 years	196	7	11	13	16	91	6	10	13	17
	≥85 years	247	5	9	11.6	15	65	3.8	8	11	16
Males	60–64 years	19	8	9.2	10.8	13.2	310	13	14	16	18
	65–69 years	50	11	14.6	18	20	366	12	14	16	19
	70–74 years	57	10	12.4	15	18	290	11	13	15	18
	75–79 years	55	9	12	13.4	15.2	184	10	12	14	16
	80–84 years	55	7.8	11	14	16	130	8.8	11	13	17
	≥85 years	110	5.8	12	13	16	102	7	10	13	15

CERAD delayed word-list recall											
	Age	No formal education					Formal education				
		Number	20^th^	40th	60th	80th	Number	20th	40th	60th	80th
Females	60–64 years	85	2	3	4	5	389	3	4	5	6
	65–69 years	237	2	3	4	5	347	3	4	5	6
	70–74 years	208	2	3	4	5	198	2	3	4	5
	75–79 years	188	1	2	3	5	149	2	3	4	5
	80–84 years	196	1	2	3	5	91	0	2	3	4
	≥85 years	247	0	1	3	4	65	0	1	3	4
Males	60–64 years	19	1	3	3	5	310	3	4	5	6
	65–69 years	50	2	4	5	6	366	3	4	5	6
	70–74 years	57	2	3	4	5	290	2	4	4	5
	75–79 years	55	1	3	4	5	184	2	3	4	5
	80–84 years	55	1	3	4	5	130	1	2	4	5
	≥85 years r	110	0	2.4	3.6	5	102	0	2	3	5

Categorical verbal fluency – Animal naming											
	Age	No formal education					Formal education				
		Number	20^th^	40th	60th	80th	Number	20th	40th	60th	80th
Males	60–64 years	85	4	7	9	13	389	8	9	11	13
	65–69 years	237	6	8	10	13	347	7.6	9	10	13
	70–74 years	208	6	9	10	13	198	6	8	10	12
	75–79 years	188	5	7	9	12	149	6	8	10	12
	80–84 years	196	5	7	9	11.6	91	5	7	8.2	10.6
	≥85 years	247	4	6	8	11	65	4	6	8	10
	60–64 years	19	3	7	9	14	310	8	10	12	15
	65–69 years	50	7	9	12	15.8	366	8	10	12	15
	70–74 years	57	6	10	12.8	16	290	8	10	12	15
	75–79 years	55	6.2	9.4	12	15	184	7	10	11	12
	80–84 years	55	6	8	10	15	130	7	8.4	10	13
	≥85 years	110	5	8	10	15.8	102	5	9	10	13

CERAD: Consortium to Establish a Registry for Alzheimer’s Disease. Bold values represent quintiles.

Table 4 displays the mean and SD for delayed recall, animal naming, and immediate recall, all broadly normally distributed. The total IDEA cognitive screen score was non-normally distributed.

**Table 4.** Mean and standard deviation values for individual items of the Identification of Dementia in Elderly Africans cognitive screen by the categories of age, sex, and education.

Education level	Sex	Age	Recall first trial		Recall second trials		Recall third trials		Animal naming		Delayed word-list recall	
			Mean	SD	Mean	SD	Mean	SD	Mean	SD	Mean	SD
No formal schooling	Females	60–64 years	3.7	2.006	4.7	1.983	4.8	2.069	8.8	5.794	3.4	2.078
		65–69 years	4.1	1.616	5.0	1.618	5.5	1.517	9.8	4.514	3.7	1.759
		70–74 years	3.8	1.710	4.5	1.636	5.1	1.818	9.9	4.156	3.3	1.817
		75–79 years	3.4	1.792	4.1	1.802	4.5	1.902	8.6	3.993	3.0	1.917
		80–84 years	3.2	1.931	4.0	1.951	4.2	2.079	8.3	4.284	2.8	1.955
		85 years and above	2.6	1.769	3.4	2.048	3.8	2.371	7.5	4.695	2.2	2.100
	Males	60–64 years	2.9	2.079	3.3	2.077	3.9	1.985	8.9	5.547	2.8	1.951
		65–69 years	4.4	2.090	5.3	1.906	5.6	2.260	11.1	5.211	4.1	2.165
		70–74 years	3.8	1.722	4.8	1.575	5.4	1.811	11.4	5.357	3.7	1.901
		75–79 years	3.6	1.470	4.4	1.532	4.5	1.824	10.9	4.550	3.3	2.082
		80–84 years	3.2	1.663	4.0	1.915	4.4	2.224	9.7	5.041	3.2	2.035
		85 years and above	3.0	1.812	4.0	2.250	4.2	2.362	9.8	5.674	2.9	2.042
Formal schooling	Females	60–64 years	4.2	1.717	5.3	1.628	5.9	1.694	10.6	4.126	4.4	1.838
		65–69 years	4.0	1.685	5.2	1.684	5.6	1.866	10.0	3.677	4.3	1.990
		70–74 years	3.7	1.699	4.7	1.859	5.2	1.969	9.3	3.731	3.6	1.882
		75–79 years	3.6	1.725	4.4	1.673	4.8	1.789	9.0	3.892	3.4	1.838
		80–84 years	3.1	1.945	3.9	2.152	4.2	2.489	7.8	3.543	2.6	1.982
		85 years and above	2.5	2.078	3.5	2.278	3.8	2.622	7.2	4.680	2.2	2.101
	Males	60–64 years	4.1	1.536	5.3	1.358	5.9	1.530	11.8	4.333	4.4	1.682
		65–69 years	4.1	1.712	5.3	1.568	5.9	1.646	11.7	4.513	4.3	1.764
		70–74 years	3.7	1.748	4.9	1.768	5.4	1.864	11.5	5.160	3.8	1.898
		75–79 years	3.4	1.709	4.6	1.509	4.9	1.691	10.2	3.679	3.5	1.771
		80–84 years	3.3	1.857	4.3	1.948	4.6	2.300	9.9	4.588	3.1	2.106
		85 years and above	3.1	1.908	3.7	2.093	4.1	2.254	9.2	4.599	2.6	2.058

SD: standard deviation.

Table 5 shows the linear regression models looking at the association between IDEA score items and age, sex, and formal schooling. In general, age was the strongest predictor across all items.

**Table 5.** Regression models for individual Identification of Dementia in Elderly Africans cognitive screen items.

		Parameter estimate	Lower 95%CI	Upper 95%CI	Significance
Recall first trial	Age	-0.049	-0.055	-0.043	<0.001
	Male	0.079	-0.035	0.192	0.173
	Formal schooling	0.014	-0.109	0.137	0.823
Recall second trial	Age	-0.058	-0.064	-0.052	<0.001
	Male	0.177	0.063	0.292	0.002
	Formal schooling	0.184	0.059	0.308	0.004
Recall third trial	Age	-0.066	-0.073	-0.059	<0.001
	Male	0.158	0.034	0.283	0.013
	Formal schooling	0.264	0.129	0.399	<0.001
Animal naming	Age	-0.092	-0.107	-0.076	<0.001
	Male	1.656	1.369	1.943	<0.001
	Formal schooling	0.178	-0.134	0.490	0.263
Delayed recall	Age	-0.063	-0.070	-0.056	<0.001
	Male	0.209	0.085	0.332	0.001
	Formal schooling	0.300	0.165	0.434	<0.001

95%CI: 95% confidence interval.

In those with a diagnosis of dementia in Tanzania (n=105, identified during the parallel epidemiological study), the mean IDEA score was 4.3 (SD: 3.370) and median 5 (0–14). Values for those with dementia in Nigeria were not available, and dementia could not be excluded in those not assessed in this two-stage study in Tanzania.

The data for word list such as immediate recall, delayed recall, and animal naming (i.e., mean and SD) are compared with the normative data obtained in a range of LMIC urban and rural settings as part of the 10/66 dementia collaboration (Supplementary Data Tables 1, 2 and 3). Animal naming and delayed recall values are lower than almost all other settings. Interestingly, values for word list-learning are higher than in some rural settings and similar to those obtained in Latin America and urban settings.

## DISCUSSION

We presented the normative data for cognitive screening in SSA. Our data emphasize the importance of considering educational level as a potential confounding variable in settings with a low background level of formal education. Since the prevalence of dementia is known to be higher in people with low education levels,^39,41,42^ these data do not suggest that the IDEA is educationally biased. In fact, previous studies by our team have indicated that this is not the case.^19,20^

The observation that scores were similar in rural Tanzania and semi-urban Nigeria is encouraging and suggests that the screening items are suitable for such populations in West and East Africa. This is perhaps unsurprising, given that the individual items within the IDEA cognitive screen were adapted from other screening tools designed for low-income and low-education settings.

We considered excluding participants who were known to have dementia. However, we felt that this would unnecessarily bias our data toward those obtaining higher cognitive scores and so be less representative. Since various categories of non-dementia cognitive impairment exist (e.g., mild cognitive impairment (MCI), ‘cognitive impairment,no dementia’ CIND), the confounding effects of clinical cognitive decline would remain. The approach to this issue remains a subject of some debate. The 10/66 cross-cultural international research group excluded individuals with known dementia from the published normative data.^24^ Similarly, the MoCA normative data were derived from an “ultra-normal” sample where potentially confounding causes of cognitive impairment were carefully screened out.^43^ The subsequent normative studies of the MoCA in other, broader, populations of older adults have indicated lower normative scores, and a lower cut-off has been widely advocated, indicating that the use of the “ultra-normal” samples leads to falsely highly published cut-off scores and the potential for high levels of false positives.^44^ We advocated that, particularly in SSA where numbers of specialist clinicians are few, and self-report of chronic diseases may be inaccurate due to limited healthcare access, a more valid and practicable approach is to represent the values seen across the spectrum of the population, an approach utilized for the normative data for other very widely used tools such as the MMSE.^45^ The fact that the range of scores obtained by those known to have dementia generally falls within ≤1.5 SDs of the mean, supports our approach.

The normative scores for categorical verbal fluency (i.e., animal naming) were substantially lower than seen in HICs and in other LMIC within the 10/66 group. Only in India, scores were comparable (i.e., 7.7–9 animals named in those without formal education)^24^ (Supplementary Data Table 1). Categorical verbal fluency appears substantially less affected by the education level than a letter or phonemic verbal fluency.^2,7,8,25^ However, verbal fluency is known to be affected by education in general,^46^ and such differences are relative. Given the score range in animal naming is noted, it would have been ideal to assess the cognitive strategies of clustering and switching within this task, reflective of frontal lobe function and considered necessary to achieve a high overall score.^46^ The frequency and breadth of animals listed would have assisted in indicating if only local familiar animals were mentioned by these older adults in SSA, who may not have the knowledge of animals found in other regions of the world. Populations with higher background levels of education may have the knowledge of animals from other sources, for example, published media. The assessment of these elements was not practicable within our study design, and we elected to focus on the accurate recording of absolute numbers. The addition of an additional categorical verbal fluency task (e.g., fruits and vegetables) would have allowed a useful comparator, though these tests are less widely used.^46^

Delayed recall was less markedly affected by education than most of the other tests. However, the data from other studies suggest that education does still impact on performance.^47^ Nevertheless, immediate recall, tested through list-learning, appears more affected by education than delayed recall in other LMIC settings, and this supports our observations.^24^ There is a well-evidenced effect of literacy and formal education on working memory ability seen across cultures,^48^ but it is not clear if this is the reason for the higher list-learning performance observed in those with higher educational levels. Performance on the CERAD list-learning task was similar to that seen in samples of older people in other LMIC settings by the 10/66 collaboration but relatively high compared with the other elements of cognitive performance (Supplementary Data Tables 2 and 3). Although the reasons for this are not clear, this illustrates the need for culture- and setting-specific normative values when assessing cognitive impairment in LMICs.

The data collected indicate that if clinicians were to use a 1.5 SD cut-off for screening of MCI, a cut-off of approximately 8/30 for word list-learning and approximately 1/10 for verbal delayed recall may be a useful “rule-of-thumb” for further assessment and/or referral. The very low scores range for animal naming may pose difficulties for screening MCI and differentiating MCI and dementia in this setting.

A strength of our study was the low level of refusals indicating that values were likely to be representative of the wider population in both settings. The use of community healthcare workers (i.e., with training and supervision) to undertake screening adds to the validity of our findings, given the shortage of specialist mental health workers across SSA. The use of computer tablets allowed a high degree of standardization and reduced data input errors and missing values.

A limitation was the inability to formally measure sensory impairment, a significant issue, given the high prevalence of uncorrected sensory impairment in SSA. This issue is likely to be equally problematic in other similar SSA settings.

A further limitation is that findings should be interpreted with caution as the inclusion of some individuals meeting dementia criteria within the sample may have resulted in lower normative scores than expected.

This is the first study reporting the normative data for the IDEA cognitive screen, previously validated for dementia screening in low-literacy populations in SSA.

Overall performance in categorical verbal fluency and delayed recall in older populations within two SSA settings was substantially lower than published norms from both HIC and LMIC settings. Despite some individuals with dementia being included, this emphasizes the need for the appropriate local normative data to be used when assessing cognitive function. We recommend that further normative data are to be collected to take into account the urban/rural differences in SSA and to consider the effect of comorbidities in hospital settings alongside the effect of sensory impairment. Larger normative datasets will allow these comparisons.

Cognitive screening performance appears to be influenced by age, education, and gender in older adults in East and West Africa, and these factors should be considered when screening for cognitive disorders. In this study, we presented the normative data that may assist clinicians in making initial decisions about the presence of cognitive impairment in different age categories by level of education, though these should be independently replicated.

Further analysis of the IDEA cognitive screen, for responsiveness to change in cognitive function over time and practice effects, would be useful to inform future interventional studies. Future larger normative datasets such as more diverse settings are recommended. It is likely, however, that over time, the educational level in older adults in SSA will improve as younger populations with more access to educational opportunities start to age. The normative data for tools currently considered educationally biased in SSA elders such as the MoCA could be considered necessary in the future.
